# Editorial to Special Issue “Glioblastoma: Recapitulating the Key Breakthroughs and Future Perspective”

**DOI:** 10.3390/ijms24032548

**Published:** 2023-01-29

**Authors:** Amit Sharma, Hugo Guerrero-Cázares, Jarek Maciaczyk

**Affiliations:** 1 Department of Stereotactic and Functional Neurosurgery, University Hospital of Bonn, 53127 Bonn, Germany; 2 Department of Neurological Surgery, Mayo Clinic, Jacksonville, FL 32224, USA; 3 Department of Surgical Sciences, Dunedin School of Medicine, University of Otago, Dunedin 9016, New Zealand

Glioblastoma (GBM) remains the most common and aggressive malignant primary brain tumor. Despite several therapeutic advances toward a broader understanding of the complex interplay of (epi)genome and the host immune system, the survival improvement in GBM patients appears to have plateaued, and relapses remain the greatest clinical challenge. Our Special Issue, “Glioblastoma: Recapitulating the Key Breakthroughs and Future Perspective”, includes 31 articles that delve into current diagnostic, therapeutic and experimental models in GBM.

The studies include the work by Drongitis et al., who examined the contribution of the chromatin remodeling gene KDM5C by stratifying GBM patients into two subgroups (KDM5C^Low^ and KDM5C^High^). Moreover, they extensively studied hypoxia-related markers to suggest that HIF1A–KDM5C is a new and relevant cancer axis in GBM [1]. Carcelén et al. also investigated hypoxia/hypoxia-inducible factors (HIF). In particular, the authors showed that HIF2α acts as a transcriptional regulator of the migration factor ODZ1 in response to hypoxia, unveiling a new signaling pathway as a potential target to prevent migration of Glioma Stem Cells (GSCs) into non-hypoxic microenvironments that may lead to tumor recurrence [2]. In the context of hypoxia, Degirmenci and colleagues discussed clinically relevant irradiation models, presenting that DNA damage response (DDR) kinases, such as Chk1, were effective in cells that escaped irradiation. However, the efficacy of such irradiation models would decrease under hypoxia [3]. Because EGFR (epidermal growth factor receptor) is recognized as an attractive target for GBM treatment, several notable investigations regarding EGFR-based novel therapeutic approaches are included. Zeneyedpour et al. focused on screening tumor-associated phospho-sites that may trigger immunoreactions and elicit autoantibodies with a notable likelihood of being detectable in the plasma. To this end, the authors introduced the novel antibody-peptide binding assay and validated it by targeting EGFR in high-grade gliomas [4]. Because EGFR amplification is often linked to more than 50% of GBM cases, Choi and colleagues evaluated the action of a molecularly targeted drug (GZ17-6.02) for its growth inhibition of GBMs expressing a specific form of EGFR, namely EGFRvIII [5]. Interestingly, Zalcman et al. addressed whether EGFR signaling is involved in androgen receptor (AR) activation in GBM. The authors extensively investigated the EGFR–AR axis and assumed that inhibiting EGFR-induced AR signaling would be beneficial [6].

As it is now well established that GSCs contribute to tumor resistance and recurrence, several authors reported their interesting findings on stem cell-focused studies. Aretz et al. investigated the effect of β-catenin (as the Wnt/β-catenin pathway is essential in GSCs) on monocyte attraction to GBM cells and further hypothesized that interactions between β-catenin and CCL2 contribute to the maintenance of GSCs through the modulating immune cell interaction and promoting GBM growth and recurrence [7]. As in Aretz et al., Dery et al. also utilized chemoattractants to strongly attract GBM neoplastic cells. Moreover, Dery et al. employed a biodegradable hydrogel (GlioGel) loaded with CXCL10, CCL2 and CCL11 to test their effects on in vitro/in vivo GBM models [8]. To address the problems related to GSCs, Seifert et al. also reported that PIM1 inhibition impacts the behavior and survival of GSCs, suggesting that PIM1 targeting may be a potential therapy against GBM [9]. The authors demonstrated that PIM1 inhibition negatively regulates the protein expression of the stem cell markers CD133 and Nestin in GBM cells. In contrast, CD44 and the astrocytic differentiation marker GFAP were up-regulated. Okada and colleagues also evaluated the therapeutic role of folate antagonists on GSCs, primarily by testing their selective cytotoxic effects in vitro and evaluating their inhibitory effects on GSCs in vivo [10]. The authors showed that GSCs were very sensitive to folate antagonists (e.g., MTX and PEM) and the expression level of folate transporter protein RFC-1 was higher in GSCs.

Several studies also addressed cellular and molecular aspects that expanded our knowledge about the mechanism characterizing this tumor, further enriching our therapeutic arsenal. For instance, owing to the strong indications from the literature supporting the involvement of the inflammatory marker Cyclooxygenase-2 (COX-2) in the Temozolomide (TMZ) resistance of GBM, Lombardi et al. evaluated the ability of the TMZ to influence COX-2 expression by considering adjuvant treatment with COX-2 inhibitors (NS398, alone or combined with TMZ) for rescuing resistant GBM patients [11]. Concurrent with TMZ-related concerns, Larrouquère et al. preclinically evaluated an attractive in vitro agent, sodium selenite (SS), for chemotherapy/cancer therapy against GBM [12]. Concerning patient survival, Santoni et al. expanded their previous findings on the role of TRPML1 channels affecting overall survival in GBM patients by demonstrating that TRPML1 and TRPML2 channels are differentially expressed in GBM and that their loss in GBM cells results in the acquisition of a more aggressive phenotype [13]. Morelli and colleagues further determined the expression of TRPML2 in several GSC lines and found a strong association of elevated expression levels with TMZ resistance in patient-derived GSCs [14].

Interestingly, for the first time, Fuentes-Fayos et al. characterized somatostatin receptor subtype 5’s (sst5TMD4) expression and the pathophysiological role of its truncated splice variant in GBM [15]. The authors reported that sst5TMD4 was significantly overexpressed in human GBM tissues and was notably associated with poor overall survival and recurrent tumors in GBM patients. By including the impact of pasireotide (a somatostatin analog), the study reveals its potential utility as a novel diagnostic/prognostic biomarker and a putative therapeutic target in GBM. Concerning therapeutical response, the article by Scherschinski et al. reports on the regulation of receptor tyrosine kinase AXL (RTK–AXL) in response to therapy and its role in GBM tumor progression [16]. These authors imply that RTK–AXL plays a critical role in both intrinsic and acquired resistance to standard GBM therapy, and monitoring their expression and shedding products as indicators of therapy resistance and tumor progression could be a promising approach for individualized therapy. Additionally, an intriguing study by Moresi and colleagues describes a highly relevant approach to identifying the proteomic markers of different anatomical locations in GBM. The authors described specific classes of proteins, especially ND (not detected) in brain proteins possibly related to the blood–brain barrier, raising the possibility of investigating unexplored mechanisms related to tumor initiation and progression [17]. Such new dimensions were also reflected in the study by Zolotovskaia et al., who performed analyses focused on identifying biomarker genes to better assign gliomas into specific subtypes. Using specific algorithms, the authors conclude that the algorithmically reconstructed genetic pathways they identified can be considered a class of next-generation biomarkers for more accurate diagnosis of gliomas [18].

As with the research articles, we also included comprehensive reviews discussing past knowledge and future perspectives for the GBM. Particularly in the context of immunotherapy in GBM, Sener et al. highlighted prior studies with checkpoint inhibitors, CAR T-cell therapy, vaccine-based therapies, viral therapies, and cytokine-based approaches focused on using the molecular composition of tumor cells to guide treatment selection [19]. On the other hand, Yutao et al. discussed the current strategies of natural killer T (NKT) cell therapy for GBM, based primarily on in vitro/in vivo experiments and clinical trials. They propose that adjuvant NKT immunotherapy with invariant NKT (iNKT) cells and cytokine-induced killer (CIK) cells may improve the clinical scenario of this devastating disease [20]. Similar to lone CIK cells, DC–CIK therapy has evolved into a widely used adoptive cellular immunotherapy. Pinho et al. described an interesting case of near-complete remission of a GBM patient treated with an allogeneic dendritic cell-based vaccine [21]. Rodriguez-Camacho and colleagues also provided a very insightful and comprehensive review of the state-of-the-art and current guidelines for treating GBM [22]. Moreover, the authors provide a future perspective on the management of the neoplasm, including the most recent therapeutic approaches in immunotherapy, e.g., new synthetic molecules, natural compounds and delivery methods, as a next step in GBM research. Given the great interest in the role of glycan–lectin interactions in the mechanisms of immunosuppression occurring in tumor immune escape, Pace et al. summarized the recent knowledge of cell surface glycans/lectins as the drivers of immunosuppression in GBM [23]. The authors discussed state-of-the-art immunosuppressive cell subsets, glycosylation changes and lectins as possible factors involved in immunosuppressive mechanisms and as potential targets for GBM treatment.

Among the multiple molecular subtypes of GBM, the mesenchymal subtype accounts for approximately 35% of all adult high-grade gliomas and is mainly characterized by loss/dysregulation of the Neurofibromatosis type 1 (NF1) gene. In this context, Scheer et al. described the mutational perspective of this particular gene and the possible impact on the neurofibromin domains along with the molecular function of its respective protein in GBM [24]. Cornelison et al. described a relatively less characterized oncogenic actin-binding protein, AVIL (advillin), as a potential therapeutic target for GBM [25]. In particular, the authors relate AVIL overexpression in GBM and its role in mediating tumor progression and metastasis through the FOXM1 and LIN28B pathways. Likewise, Gonzalez-Mora addressed estrogen receptors (ER), the relevance of ER isoforms and potential drugs that could be used in endocrine therapy (hormone receptor agonists or antagonists) for the treatment of high-grade gliomas [26].

Among existing biomarkers and future possibilities of targeting pathways in GBM, Senhaji et al. provided an overview of the molecular and circulating biomarkers necessary for diagnosing and monitoring GBM [27]. In addition to providing information on new advances in GBM therapy, the authors also highlighted the importance of integrating all these known markers as valuable tools for improving patient care. Moreover, Rocchi et al. reviewed protein quality control (PQC) and focused on the Bcl-2-associated athanogene (BAG)-family, with special emphasis on the activity of Bag3-HSP70 towards cancer propagation, and encouraged further understanding and targeting Bag3 for consideration as a potential option for GBM treatment [28]. Based on the altered expression and distinctive features in tumor growth, Manzano et al. suggested that C3G (guanine-nucleotide releasing factor binding to the SH3 domain of Crk, encoded by the RAPGEF1 gene) could represent a new biomarker for GBM diagnosis, prognosis and personalized treatment of patients in combination with other molecular markers of GBM [29]. Since numerous signaling pathways have been implicated in gliomas, including the Hippo pathway, Casati et al. discussed in depth the activation (in relation to chemoresistance and tumor immunosuppression) of this particular pathway in GBM, its cross-linking with other important signaling pathways and its being a promising target for the treatment of high-grade gliomas [30]. Interestingly, in the context of drug delivery, Pena et al. reviewed current polymer and formulation methods used for interstitial therapy and further addressed the development and limitations of Gliadel^®^, the first and only FDA-approved interstitial therapeutic for GBM [31]. In addition, the authors provide considerations for the rational design of future implantable biodegradable materials along with the application of combination therapy to further enhance current research efforts.

Collectively, we are convinced that the original research articles and reviews published in our Special Issue will undoubtedly enhance our understanding of the pathogenesis and novel therapy options of GBM. Considering the plethora of information provided throughout this Special Issue, it is reasonable to propose that GBM patients can express new hope for innovative treatment. However, much work remains to be done.

## Data Availability

Not applicable.
